# Event-Related Potentials in Relation to Risk-Taking: A Systematic Review

**DOI:** 10.3389/fnbeh.2018.00111

**Published:** 2018-06-19

**Authors:** Dilushi Chandrakumar, Daniel Feuerriegel, Stefan Bode, Megan Grech, Hannah A. D. Keage

**Affiliations:** ^1^Cognitive Ageing and Impairment Neurosciences Laboratory, School of Psychology, Social Work and Social Policy, University of South Australia, Adelaide, SA, Australia; ^2^Decision Neuroscience Laboratory, Melbourne School of Psychological Sciences, University of Melbourne, Melbourne, VIC, Australia

**Keywords:** risk, ERP, event-related potentials, P3, FRN, ERN, MFN

## Abstract

Event-related potentials (ERPs) have been used to investigate neural mechanisms underlying risk-related decisions over the last 16 years. We aimed to systematically evaluate associations between risk-taking and ERP components elicited during decisions and following feedback. A total of 79 articles identified from PsychINFO and PubMed databases met the inclusion criteria. Selected articles assessed early ERP components (feedback-related negativity/FRN, error-related negativity/ERN, and medial frontal negativity/MFN) and the mid-latency P3 component, all using gambling paradigms that involved selecting between choices of varying risk (e.g., Iowa Gambling Task, Balloon Analogue Risk Task, and two-choice gambling tasks). The P3 component was consistently enhanced to the selection of risky options and when positive feedback (as compared to negative feedback) was provided. Also consistently, the early negative components were found to be larger following feedback indicating monetary losses as compared to gains. In the majority of studies reviewed here, risk was conceptualized in the context of simple economical decisions in gambling tasks. As such, this narrow concept of risk might not capture the diversity of risky decisions made in other areas of everyday experience, for example, social, health, and recreational risk-related decisions. It therefore remains to be seen whether the risk-sensitivity of the ERP components reviewed here generalizes to other domains of life.

## Introduction

Risk is defined as uncertainty of an outcome when presented with multiple options with variable outcomes (e.g., harm or loss), when the outcome probabilities of the possible outcomes are unknown (Mohr et al., 2010; Euser et al., 2011; Kóbor et al., 2014). Risk-taking is the tendency to knowingly engage in behavior with potentially undesirable outcomes (Boyer, 2006). In the economic domain, for example, selecting to gamble large (as opposed to small) monetary quantities can result in gaining large monetary rewards, but could also lead to large monetary losses. In this example, individuals selecting to gamble the large monetary quantities are considered to be engaging in risky decision-making. When making a risky decision, one considers the risk-benefit trade-off between the perceived risks and perceived benefits of selecting between choices (Blais and Weber, 2006). Everything we do has some inherent risk, and poor risk-related decision-making has significant impacts not only economically, but also more broadly. For example, people might risk acute injury when they engage in (highly rewarding) extreme sports, or they risk chronic illnesses when they smoke or take recreational drugs. Most everyday decisions also require risk-related decision-making, for example when navigating through critical traffic situations, or in social conflicts with co-workers or friends.

The neural mechanisms underlying risk-related decisions have been investigated using event-related potentials (ERPs), which index neural activity time-locked to an event of interest. This event may be the presentation of a stimulus, which provides options with different levels of risk attached, or the execution of a response, indexing decisions associated with more or less risk. But more commonly investigated are ERP responses to feedback from risky decision-making, where ERP responses are influenced by whether the feedback outcome is positive or negative. Although risky decision-making can be measured using behavioral responses to risk-related stimuli alone, ERPs provide additional direct and temporally precise measurements of neural processes involved in decision-making (Euser et al., 2011).

Early components of the ERP such as the feedback-related negativity (FRN), error-related negativity (ERN), and medial frontal negativity (MFN), along with the mid-latency P3 component, have been investigated extensively in relation to risk-taking. Other ERP components, the reward positivity being one (e.g., Holroyd et al., 2011) has been investigated relative to risky decision-making, and the P1 and P2 components, relative to general stimulus processing (e.g., Nelson et al., 2011; Lole et al., 2015). However the reward positivity, P1, P2 components have been studied far less frequently compared to the FRN, ERN, MFN, and P3 components, and will not be the focus of this review. Throughout the review, the term P3 will be used to refer to the P3b component.

The FRN, ERN, MFN, and P3 components are commonly elicited during tasks that involve risk-taking, as well as when processing task-related feedback following risk-related decisions (Christie and Tata, 2009; Gu et al., 2010; Hassall et al., 2013; Sambrook and Goslin, 2015). Previous ERP studies of risk-related decision-making have investigated economical decision-making using gambling paradigms (Kamarajan et al., 2009, 2010; Leng and Zhou, 2010) such as the Iowa Gambling Task (Zottoli and SutherlandS, 1994), the Balloon Analogue Risk Task (Lejuez et al., 2002), and various forms of two-choice gambling tasks (Ma et al., 2011; Rao et al., 2013). All of these tasks involve some form of risky decision-making as participants are required to select from a number of available options, which comprise possible gain or loss outcomes. These components are time-locked to the onset of feedback in risky decision-making tasks.

The ERN is a negative-going deflection, peaking between 80–100 ms post response onset and around 250 ms from feedback stimulus onset at medio-frontal sites (Falkenstein et al., 1990; Gehring et al., 1990; Holroyd et al., 2003; Donkers et al., 2005; Ullsperger et al., 2014). The ERN is enhanced following erroneous task-related responses (i.e., following an incorrect response) and feedback stimuli signaling response errors. The FRN and MFN are also negative deflections, peaking 250–300 ms post feedback onset at medio-frontal sites (Miltner et al., 1997; Gehring and Willoughby, 2002). These early components partially index reward-related neural activity, such that more negative components are elicited in response to negative feedback than positive feedback when performing a reward-related task. For example, the task goal in a gambling task is to maximize rewards. Loss feedback signals a violation of this goal, as the expectation is for positive feedback as compared to negative feedback (Oliveira et al., 2007), resulting in a larger ERP response to negative compared to positive feedback (Gehring and Willoughby, 2002). Outside the risk-related area, the FRN appears to be larger (more negative) for surprising events (Hauser et al., 2014), and the ERN is implicated in the detection of endogenous response errors (Yeung et al., 2004), suggesting that these early negative ERP components are broadly related to conflict monitoring. The amplitudes of these components are also influenced by reward magnitude; larger reward-related outcomes elicit larger (more negative) component amplitudes (Ullsperger et al., 2014; Sambrook and Goslin, 2015). Typically, these components are larger following negative as opposed to positive feedback (e.g., see Euser et al., 2011 for FRN; Goyer et al., 2008 for MFN; Hewig et al., 2007 for ERN).

The FRN/ERN/MFN components are often referred to interchangeably in the literature, however they do slightly differ: the ERN more closely indexes endogenous response errors (response ERN) and violations of reward-related expectations (i.e., reward prediction error; feedback ERN); the FRN and MFN are more closely associated with feedback valence and feedback magnitude (Masaki et al., 2006; Gu et al., 2010). In the current review, the term *early error-detection components* will be used to refer to the FRN/ERN/MFN. This is because these components are often not explicitly differentiated in studies investigating risk-related decision-making, giving rise to discrepancies in the terminology used to describe these highly interrelated ERP components.

The P3 ERP component is defined as a positive deflection between 300–600 ms following stimulus onset, commonly maximal in amplitude at centro-parietal electrodes (Picton, 1992; Polich, 2007). There are several competing views on the cognitive underpinnings of the P3, based on concepts such as working memory updating, stimulus categorization, strategic processing, and evidence accumulation in perceptual decision-making (Donchin, 1981; Verleger et al., 2005; Yuan et al., 2007; O'Connell et al., 2012). This component is also thought to index the evaluation of the task-relevance of incoming stimuli, where a larger P3 amplitude is observed for stimuli that are perceived as being more salient or relevant to task goals (Nieuwenhuis et al., 2005).

The emotional valence of a stimulus, which refers to the perception of attraction or aversion toward a stimulus, influences the P3, such that stimuli with high positive or negative emotional valence produce larger P3 amplitudes than neutral stimuli (Johnston et al., 1986). The P3 is also affected by expectation (i.e., the violation or fulfillment that a given stimulus will appear), hence, unexpected or rare stimuli (e.g., in oddball paradigms) elicit more pronounced P3 amplitudes than expected stimuli (Donchin, 1981). These association of the P3 with experimental manipulations has led to the hypothesis that the P3 indexes general mechanisms involved in the processing of surprise and belief-updating (Endrass et al., 2016; Kardos et al., 2016).

**Figure 1:**
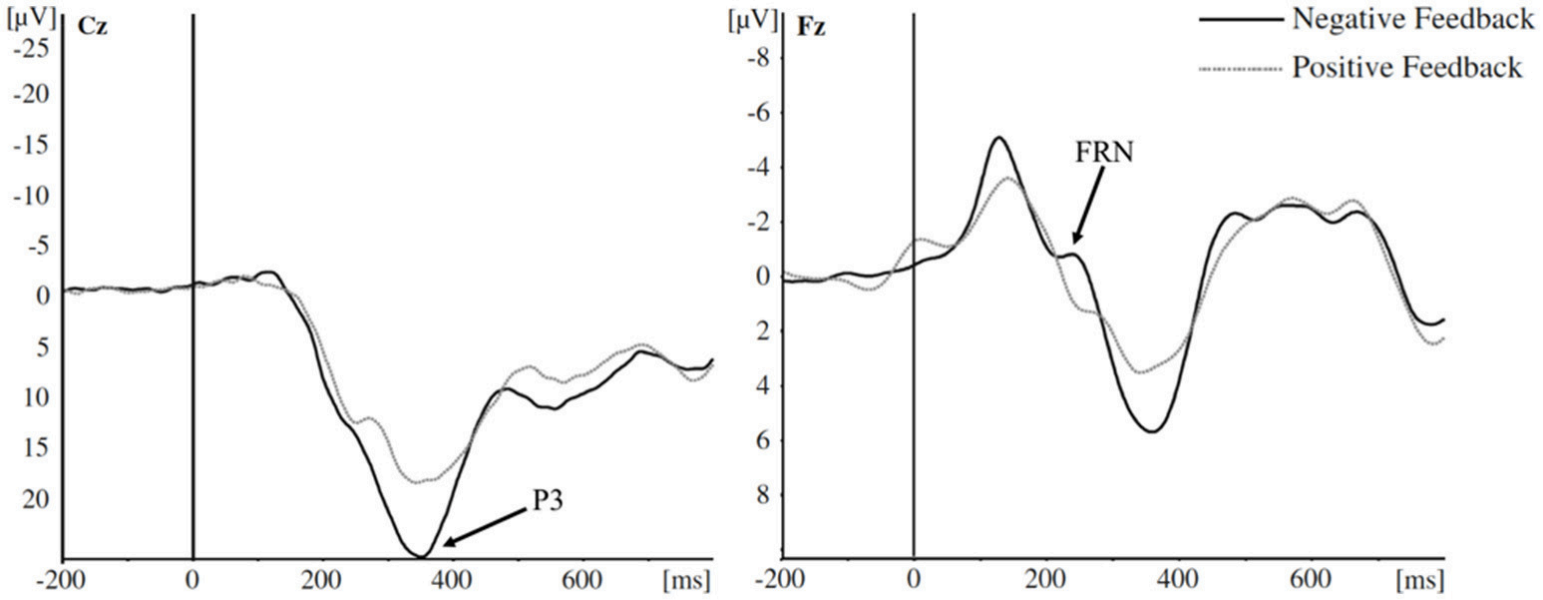
Example P3 and FRN ERP waveforms. In this example, more positive-going P3 amplitudes are elicited to negative (compared to positive) feedback between 300 and 400 ms at electrode Cz, and more negative-going FRN amplitudes are elicited to negative feedback between 200 and 280 ms at electrode Fz Image adapted from Euser et al. (2011). Image licensed under a Creative Commons Attribution Noncommercial License.

So far, there has been no systematic evaluation of risk-related decisions and ERP components across tasks. Sambrook and Goslin (2015) carried out a meta-analysis on selected studies (i.e., not systematic) of the FRN with respect to reward prediction only, and Walsh and Anderson (2012) also reviewed selected studies reporting FRN and ERN results (i.e., also not systematic). These previous reviews focused solely on the FRN and ERN, hence the P3 ERP, which is also a commonly reported component, relative to risky decision-making has not been reviewed; and therefore the consistency of effects across P3 studies of risk is unknown. Refer to Figure 1 for example P3 and FRN ERP waveform (adapted from Euser et al., 2011).

The current study aimed to fill this gap: to systematically evaluate the evidence for associations between risk-related decisions and the ERP components that have been identified as relevant for risk-related decision-making, the P3 and FRN/ERN/MFN. In doing so, the secondary aim of this review was to identify how ERP component findings are associated with aspects of experimental methodology such as feedback valence, magnitude of feedback, and expectation manipulations related to risk. This review is important for two reasons. First, despite 16 years of ERP research, there has been no effort to systematically evaluate associations between ERP components in relation to risk-related decisions and feedback; this comprehensive review will guide future research methodology and point to novel areas of research for those in the risk field. Secondly, the components assessed in relation to risk-related decisions and feedback are not specific to risk. Review findings, especially those related to experimental manipulations effects, will enable those in related cognitive fields to extend more general cognitive neuroscience theories and integrate risk-related content that is often field-specific. For example, findings from fields specific to ERPs and risk-taking can be applied to understanding behavioral risk-taking; individual differences in risk sensitivity could be determined based on ERP responses, which could consequently assist with understanding why some individuals are more prone to risk-taking than others.

## Methods

### Search strategy and selection criteria

PubMed and PsychINFO databases were searched using the terms (electroencephalography OR EEG OR ERP OR event-related^*^ OR “event related^*^” OR “evoked potential^*^” OR “evoked-potential”) AND (gambl^*^ OR “risk tak^*^” OR “risk-tak^*^” OR “risk perception”) on October 29, 2017. Inclusion criteria were: healthy participants, original peer-reviewed research articles, published in English, using EEG methodology and reporting the P3 or FRN/ERN/MFN ERP components, using a cognitive paradigm involving risk (defined as active engagement in a task which involves selecting between options varying in risk), and reporting on a statistical association between any ERP component and task-related risk. Studies containing only clinical populations (i.e., no effects reported for a control group), studies with a sample size less than 10, and case studies were excluded. If available, from clinical studies, only data from healthy controls was included in the review. The search was not restricted to any time range.

**Figure 2:**
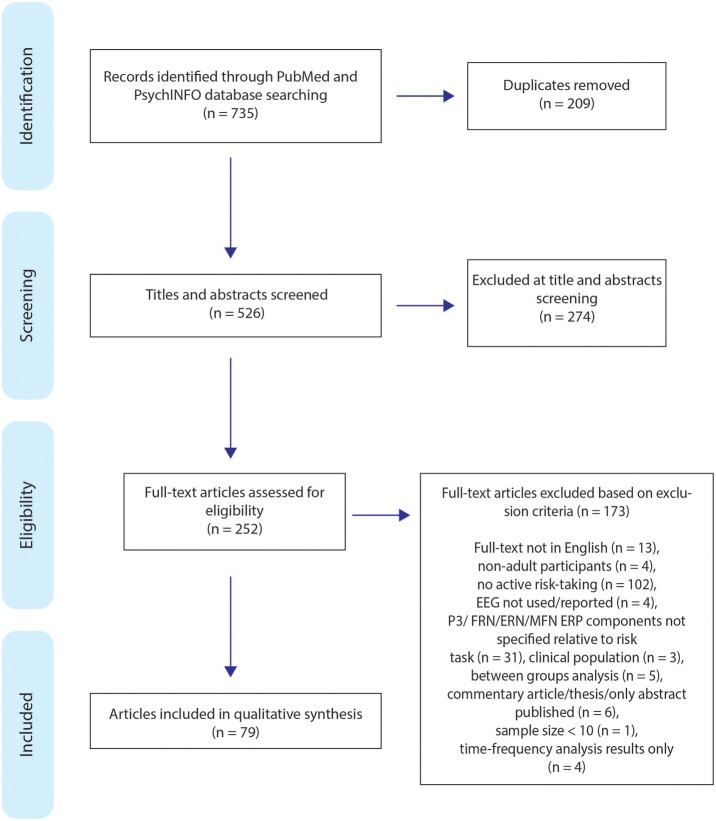
Flow chart of the search process and article selection for the systematic review (*n* = number).

This review was conducted adhering to the Preferred Reporting Items for Systematic Reviews and Meta-Analyses (PRISMA) guidelines (Moher et al., 2009). All titles and abstracts were screened, assessed for eligibility, and relevant data extracted by two individuals (DC and MG). A total of 735 articles were found; 209 duplicates were identified and removed from the search, the remaining articles were further screened for eligibility, excluding 274 articles during titles and abstract screening, resulting in 252 articles being selected to be read full-text. The review process is displayed in Figure 2.

The following information was extracted from selected articles: participant demographics (age and gender distribution), sample size, risk measure, risk operationalization, experimental task, the ERP component measured, measurement location of component, and component measurement type (peak or mean amplitude or peak latency).

## Results

After applying the exclusion criteria, 79 articles remained and these were selected for the final review. The selected articles reported either the P3 and/or an early error-detection ERP component measured during a risk-related task (following a risk-related stimulus, or in response to risk-related feedback). All studies employed a gambling task, specifically, the Balloon Analogue Risk Task (BART), Blackjack, Cued Learning Gambling Task, Fruit Gambling Task, Iowa Gambling Task (IGT), Single Outcome Gambling Task (SOG), or a Two Choice Gambling Task. A description of tasks employed in the selected studies can be found in Table 1. A summary of extracted data is displayed in Table 2.

**Table 1:** Description of gambling tasks in studies included for review.

**Task**	**Description**	**Outcomes**	***n***
Balloon Analogue Risk Task (BART)	Each trial requires selecting whether to pump a computerized balloon or collect accrued points. Pumping the balloon gains more points but is considered risky as the balloon could burst.	Points gained when pumping balloon. Points lost when balloon bursts.	5
Blackjack	The goal is to obtain a value greater than 17, without going over the value of 21. Selecting more cards when the value is close to 17 is risky.	Gain of virtual money when value is >17 and < 21. Loss when value is < 17 or >21.	3
Cued Learning Gambling Task	Cued associations are learned. Selection of a response associated with a smaller probability of win is considered risky.	Monetary gain when selecting responses associated with win cues. Loss when selecting responses associated with loss cues.	8
Fruit Gambling Task	The goal is to yield as many of the same fruit symbols in four columns as possible. Each trial requires the selection between two bet options; selection of the large bet is considered risky.	Monetary win when at least three of the symbols are the same; all other options produce a monetary loss.	3
Iowa Gambling Task (IGT)	On each trial, one of four decks of cards is selected. Two decks are advantageous and two are disadvantageous. Selection of disadvantageous decks is considered risky.	Monetary win when selecting cards from advantageous decks and monetary loss when selecting from disadvantageous decks.	11
Single Outcome Gambling Task (SOG)	Requires selecting between one of two boxes labeled “10” or “50,” each representing a monetary value. Selection of the choice with a larger monetary value is considered risky.	Monetary win or loss of 10 or 50 based on selected option.	4
Two Choice Gambling Task	Requires selecting between two monetary options. The choice of the larger monetary value is considered risky. Variations in the values used exist, with the most common being “25” and “5.”	Monetary win or loss of the value selected.	46

*n = number of studies employing the task*.

**Table 2:** Summary of studies included in the systematic review sorted by task.

**Author**	**Participant characteristics**	**P3 peak/mean amplitude (location and time window)**	**P3 major finding(s)**	**Early error-detection component peak/mean amplitude (location and time window)**	**Early error-detection component major finding(s)**
**BALLOON ANALOG RISK TASK**					
Ba et al., 2016^*^	*n* = 24 (24 M), range 22–28 years. Risky drivers *M* = 24.5, *SD* 2.2 years. Safe drivers *M* = 23.6, *SD* = 1.1 years)	Mean amplitude (Fz, FCz, Cz; 350–450 ms post feedback)	G < L	FRN mean amplitude (Fz, FCz, Cz; 200–300 ms post feedback)	G < L
Euser et al., 2011	*n* = 64 (64M), *M* = 20.5 *SD* = 2 years, range 18–25 years	Peak amplitude (Fz, FCz, Cz; 300–400 ms post feedback)	G < L	FRN peak amplitude (Fz, FCz, Cz; 200–300 ms post feedback)	G < L
Hassall et al., 2013	*n* = 14 (2M), *M* = 21.5 *SD* = 1.5 years	Peak amplitude (Cz; 300–450 ms following risky stimulus^+^)	Larger P3 for risky response compared to non-risky response selection; larger P3	–	–
Kóbor et al., 2014	*n* = 32 (4M), *M, SD* and range for age unavailable	Peak amplitude (Fz, FCz, Cz; 300–600 ms post feedback)	G < L	–	–
Mussel et al., 2015	*n* = 20 (20M), *M* = 24.5, range 20–23 years. *SD* unavailable	–	–	FRN measurement type and site not specified (270–310 ms post feedback)	G < L
**BLACKJACK**					
Hewig et al., 2007	*n* = 18 (2M), *M* = 20.9 *SD* = 2.4, range 18–26 years	–	–	ERN mean amplitude (Fz, FCz, Cz, CPz, Pz; 300–350 ms post feedback)	G < L; the greater the probability of a gain outcome, the larger the amplitude after loss outcomes
West et al., 2014a	*n* = 103 (gender unavailable), *M* = 20 *SD* = 3 years	Mean amplitude (Pz; 500–600 ms post feedback)	Busts > L > G; P3 amplitudes larger for large gains and losses than small gains and losses	FRN mean amplitude (FCz; 295–325 ms post feedback)	G < L < busts; larger amplitude for small gains/losses than large gains/losses
West et al., 2014b	*Younger adults n* = 20 (10M), *M* = 20.1 *SD* = 2.1 years. Older adults *n* = 20 (7M), *M* = 70.4 *SD* = 6.1 years. Range unavailable.	Mean amplitude (Pz; 600 ms post feedback)	Busts > G or L; P3 amplitudes larger for large gains and losses than small gains and losses	–	–
**CUED LEARNING GAMBLING TASK**					
Deng et al., 2012	*n* = 24 (8M), *M* = 22.9 *SD* = 1.5, range 20–25 years	Mean amplitude (Fz, Cz, Pz; 350–450 ms post cue presentation^∧^)	G > L	–	–
He et al., 2013	*n* = 30 (17M), range 30–50 years, *SD* unavailable	Peak amplitude (F3, Fz, F4, C3, CZ; 300–500 ms post risky stimulus)	Larger P3 for high-risk than low-risk options	–	–
Kogler et al., 2017	*n* = 23, *M* = 22.8 *SD* = 3.0 years	Mean amplitude (Pz; 300–500 ms post feedback)	G < L for expected-certain feedback; G > L for expected-uncertain and unexpected-uncertain feedback	FRN mean amplitude (FCz; 200–300 ms post feedback)	G < L (for expected-uncertain, unexpected-uncertain and expected-uncertain feedback)
Pfabigan et al., 2011a	Experiment 1: *n* = 29 (13M), *M* = 26.1 *SD* = 3.1 years Experiment 2: *n* = 26 (26F), *M* = 23.4 *SD* = 3.4 years	Peak amplitude (FCz) and peak latency 300–600 ms post feedback	Experiment 1: longer P3 latency following losses; no significant valence effects were found Experiment 2: longer P3 latency following losses;	FRN peak amplitude (FCz; 200–400 ms post feedback)	Experiment 1: G < L Experiment 2: G < L
Pfabigan et al., 2011b	*n* = 20 (10M), *M* = 26.6 *SD* = 3.3 years	–	–	FRN peak amplitude and latency (FCz; 200–350 ms post feedback)	G < L
Pfabigan et al., 2012	*n* = 26 (0M), *M* = 23.4 *SD* = 3.4, range 19–32 years	Mean amplitude (Pz; 300–600 ms post feedback)	No significant associations	–	–
San Martin et al., 2013	*n* = 41 (20M), *M* = 23.1, range 18–31 years	Mean amplitude (Pz; 416–796 ms post feedback)	G < L; Larger amplitude for large gains and losses than small gains and losses	FRN peak amplitude (FCz; 204–272 ms post feedback)	G < L
San Martin et al., 2016	*n* = 42 (22M), *M* = 22.0, Range 18–34 years, *SD* unavailable	Mean amplitude (Pz; 416–796 ms post feedback)	G < L; larger amplitude for larger gains and losses than small gains and losses	FRN mean amplitude (FCz; 204–272 ms post feedback)	G < L; larger amplitude for large gains and losses than small gains and losses
**FRUIT GAMBLING TASK**					
Lole et al., 2013	*n* = 17 (7M), *M* = 18.7 *SD* = 4.8, range 18–23 years	Mean amplitude (Fz, FCz, Cz, Pz; 472 ms post feedback)	G > L	FRN peak amplitude (Fz; 290 ms post feedback)	G < L; smaller amplitude for large gains and losses than small gains and losses
Lole et al., 2015	*n* = 20 (9M), *M* = 28.8 *SD* = 11.2, range 18–49 years	Peak amplitude (Pz; 250–600 ms post feedback)	G > L	FRN peak amplitude (Fz; 250–350 ms post feedback)	Near gains < L
Luo et al., 2011	*n* = 19 (8M), *M* = 22 *SD* = 1.6, range 19–25 years	Mean amplitude (Pz, Cz, FCz, Fz; 300–550 ms post feedback)	G > near misses > full misses	FRN mean amplitude (Pz, Cz, FCz, Fz; 200–300s post feedback)	G < near misses < full misses
**IOWA GAMBLING TASK**					
Azcárraga-Guirola et al., 2017	*n* = 19 (6M), *M* = 38.3 *SD* = 10.3 years	Mean amplitude (F3, Fz, F4, C3, Cz, C4, P3, Pz, and P4) and peak latency (Pz), 311–500 ms post feedback	G > neutral (no gain/loss); no significant latency associations	FRN mean amplitude (F3, Fz, F4, C3, Cz, C4, P3, Pz, and P4) and peak latency (Cz), 200–310 ms post feedback	G < L; G < neutral (no gain/loss) feedback; no significant latency associations
Ba et al., 2016^*^	*n* = 24 (24 M), range 22–28 years. Risky drivers *M* = 24.5, *SD* = 2.2 years. Safe drivers *M* = 23.6, *SD* = 1.1 years)	Mean amplitude (Fz, FCz, Cz; 350–450 ms post feedback)	G > L	FRN mean amplitude (Fz, FCz, Cz; 200–300 ms post feedback)	G < L
Balconi et al., 2015	*n* = 22 (12M), *M* = 23.8 *SD* = 2.6, range 19–25 years	Peak amplitude (F3, Fz, F4, Cz, C3, C4, P3, T7, Pz, P4, T8, Oz, O1, O2; 300–400 ms post feedback)	Larger amplitude for large gains and losses than small gains and losses	FRN peak amplitude (F3, Fz, F4, Cz, C3, C4, P3, T7, Pz, P4, T8, Oz, O1, O2; 150–300 ms post feedback)	Larger amplitude for large gains and losses than small gains and losses
Bianchin and Angrilli, 2011	*n* = 16 (16M), *M* = 24.6 *SD* = 3.1, range 20–30 years	–	–	FRN mean amplitude (Fz, FCz; 310–330 ms post feedback)	G < L
Christie and Tata, 2009	*n* = 19 (7M), *M* = 22 years, *SD* and range unavailable	Mean amplitude (CPz; 310–350 ms post feedback)	Larger P3 for high-risk relative to low-risk bets	FRN mean amplitude (FCz; 200–300 ms post feedback)	G < L
Cui et al., 2013	*n* = 26 (14M), *M* = 22.4 *SD* = 1.7, range 19–25 years	Mean amplitude (Cz, CPz, Pz; 300–450 ms post feedback)	Large P3 for high-risk bet in the right hemisphere and large P3 for low-risk bet in left hemisphere; larger P3 for large gains and losses than small gains and losses	FRN peak amplitude (Fz, FCz, and Cz; 220–330 ms post feedback)	G < L
Fielding et al., 2017	*n* = 36 (20M), *M* = 42.5 *SD* = 19, range 18–77 years	Mean amplitude (F3, Fz, F4, C3, Cz, C4, P3, Pz and P4; 300–400 ms post feedback)	G < L only when gain probability is 80% G < L only with 80% gain probability; no significant associations with 20% gain probability	FRN peak amplitude (FCz, CPz and Pz)	No significant associations
Giustiniani et al., 2015	*n* = 20 (10M), *M* = 38.7 *SD* = 18.3, range 21–59 years	Amplitude (mean or peak not specified; FPz, Fz, Cz, CPz, Pz, Oz; 450–500 ms post feedback)	G < L	–	–
Mapelli et al., 2014	*n* = 15 (11M), *M* = 60.7 *SD* = 9.8, range 43–77 years	Mean amplitude (Cz, Fz, Pz; 350–450 ms post feedback)	G > L	FRN mean amplitude (Cz, Fz, Pz; 250–350 ms post feedback)	G > L
Schuermann et al., 2011	*n* = 18 (2M), *M* = 27.3 *SD* = 6.6 years	Mean amplitude (CPz, Pz; 300–400 ms post feedback)	No significant associations	FRN mean amplitude (FCz, Fz; 240–310 ms post feedback)	G < L
Tamburin et al., 2014	*n* = 24 (15M), *M* = 46.1 *SD* = 17.5, range 23–71 years	Peak amplitude (Pz; 250–450 ms post feedback)	G > L	FRN peak amplitude (Fz; 150–310 ms post feedback)	G > L
**SINGLE OUTCOME GAMBLING TASK**					
Kamarajan et al., 2009	*n* = 50 (25M), range 18–25 years, *SD* unavailable	Peak amplitude and peak latency (Fz, FCz, Cz, Pz; 275–700 ms post feedback)	G > L; larger amplitude for large gains and losses than small gains and losses; shorter latency for gains than losses;	MFN peak amplitude and peak latency (Fz, Cz; 200–275 ms post feedback)	G > L; shorter latency for gains than losses
Kamarajan et al., 2010	*n* = 40 (40M), *M* = 21.1 *SD* = 3.4, range 18–35 years	Peak amplitude and peak latency (FCz, Pz; 275–700 ms post feedback)	G > L; larger amplitude for large gains and losses than small gains and losses; shorter latency for gains than losses; shorter latencies for small gains and losses than large gains and losses	–	–
Masaki et al., 2006	*n* = 20 (13M), *M* = 22.9 *SD* = 2.0, range 19–26 years	–	–	MFN peak amplitude (FCz; 250 ms post feedback)	G < L only for error outcomes
Onoda et al., 2010	*n* = 19 (5M), *M* = 23.3 *SD* = 2.7, range 19–29 years	–	–	FRN peak amplitude and peak latency (FCz; 250–400 ms post feedback)	G < L; larger amplitudes for large gains and losses than small gains and losses; no difference in peak latency between gains and losses
**TWO-CHOICE GAMBLING TASK**					
Endrass et al., 2016	*n* = 18 (5M), *M* = 27.2, *SD* = 8.2 years	Peak amplitude (Fz, FCz, CPz, Pz; 300–500 ms post feedback)	G < L	FRN peak amplitude (Fz, FCz, CPz, Pz; 200–400 ms post feedback)	G < L
Gehring and Willoughby, 2002	*n* = 12 (6M), range 19–30 years, *M* and *SD* unavailable	–	–	MFN mean amplitude (Fz; 200–300 ms post feedback)	G < L
Goyer et al., 2008	*n* = 20 (10M), *M* = 23, range 19–38 years, *SD* unavailable	–	–	MFN mean amplitude (Fz, Pz; 200–300 ms post feedback)	G < L
Gu et al., 2010	*n* = 33 (14M), *M* = 23.6 *SD* = 1.9 years	–	–	FRN peak amplitude (FCz; 200–400 ms post feedback)	G < L
Heitland et al., 2012	*n* = 60 (0M), *M* = 20.9 *SD* = 2 years	–	–	FRN mean amplitude (Fz; 240–260 ms post feedback)	G < L
Ibanez et al., 2012	*n* = 25(18M), range 18–54 years, *M* and *SD* unavailable	Mean amplitude (FCz; 372–464 ms post feedback)	G > L; larger P3 for large gains and losses than small gains and losses	fERN mean amplitude (FCz; 225–281 ms post feedback)	G < L; smaller amplitude for large and small losses compared to large gains
Kardos et al., 2016	Young adults: *n* = 18 (3M), range 18–32 years. Older adults: *n* = 17 (4M), range 62–72). *M*s and *SD*s unavailable	Mean amplitude (CP1, CPz, CP2, P1, Pz, P2; 350–400 ms post feedback)	G > L	FRN mean amplitude (F1, Fz, F2, FC1, FCz, FC2, Cz; 225–275 ms post feedback)	G < L; no effect of magnitude was observed
Kokmotou et al., 2017	*n* = 29 (15M), *M* = 22.5 *SD* = 3.6 years	–	–	FRN peak amplitude (Cz & 38; 233–263 ms post feedback)	G < L
Leicht et al., 2013	*n* = 22 (6M), *M* = 26.3 *SD* = 3.1 years, range unavailable	–	–	FRN peak amplitude (Fz; 220–300 ms post feedback)	G < L
Leng and Zhou, 2010	*n* = 28 (16M), *M* = 23.5, range 23–28 years, *SD* unavailable	Peak amplitude (Fz, FCz, Cz, CPz, Pz; 250–600 ms post feedback)	G > L	FRN peak amplitude (Fz, FCz, Cz, CPz, Pz; 200–400 ms post feedback)	G < L
Leng and Zhou, 2014	*n* = 24 (14M), *M* = 23.7, range 21–31 years, *SD* unavailable	Mean amplitude (FCz, Cz; 280–360 ms post feedback)	G > L	FRN mean amplitude (FCz, Cz; 200–280 ms post feedback)	G < L
Li et al., 2009	*n* = 16 (7M), *M* = 22.4, range 20–25 years, *SD* unavailable	–	–	FRN mean amplitude (FCz, Cz, Fz, CPz, Pz; 250–310 ms post feedback)	G < L when the loss was small and gain was large; larger amplitudes for small gains than large gains
Luo and Qu, 2013	*n* = 18 (8M), *M* = 20.8 *SD* = 1.26 years	Mean amplitude (Pz, Cz, FCz, Fz; 300–550 ms post feedback)	G > L	FRN mean amplitude (Fz, FCz, Cz, Pz; 200–300 ms post feedback)	G < L; larger amplitudes for small gains and losses than large gains and losses
Ma et al., 2011	*n* = 14 (0M), *M* = 23.0 *SD* = 3.1 years	Peak amplitude (CP1, CPz, CP2, CP4, P3, P1, Pz, P2, P4; 250–600 ms post feedback)	G > L at site Pz only	FRN mean amplitude (FCz, Cz, CPz; 280–380 ms post feedback)	G > L
Marco-Pallares et al., 2010	*n* = 96 (50M), *M* = 23.7, range 21–46 years, *SD* unavailable	–	–	FRN mean amplitude (Cz; time window unreported)	G < L
Mushtaq et al., 2015	*n* = 27 (10M), *M* = 20.9 *SD* = 2.6, range 18–30 years	Mean and peak amplitude (Pz; 350–500 ms post feedback)	G > L (mean & peak)	FRN peak amplitude (FCz; 250–350 ms post feedback)	G < L
Mushtaq et al., 2016	*n* = 27, *M, SD* and range unavailable	Mean amplitude (Pz; time window unreported)	Larger amplitude following small compared to large outcomes	FRN mean amplitude (Fz; time window unreported)	G < L
Nelson et al., 2011	*n* = 79 (42M), *M* = 25 years, *SD* and range unavailable	Peak amplitude (Cz; 296–500 ms post feedback)	G > L	FRN peak amplitude (FCz; 203–328 ms post feedback)	G < L
Nieuwenhuis et al., 2004	Experiment 1: *n* = 14 (6M), *M* = 22, range 18–24 years Experiment 2: *n* = 12 (6M), *M* = 22.4, range 18–26 years. *SD*s unavailable	–	–	ERN mean amplitude (FCz; 210–310 ms post feedback)	Experiment 1: G < L Experiment 2: only error trials showed larger amplitudes than correct trials
Oberg et al., 2011	*n* = 21, *M* and *SD* unavailable and range unavailable	Peak amplitude (Cz; 310–350 ms post feedback)	Larger P3 for high-risk than low-risk bets	FRN mean amplitude (FCz; 236–256 ms post feedback)	Feedback following high-risk outcome produced an early error-detection component, whereas this component was not observed following low-risk outcomes
Polezzi et al., 2010	*n* = 24 (7M), *M* = 23.5, range 19–41 years, *SD* unavailable	Mean amplitude (F3, Fz, F4, CP3, CPz, CP4; 300–500 ms post feedback)	G > L; larger P3 for large gains and losses than small gains and losses	FRN mean amplitude (F3, Fz, F4; 200–300 ms post feedback)	G < L
Rao et al., 2013	*n* = 15 (9M), *M* = 21.3 *SD* = 1.7 years, range unavailable	Mean amplitude (Fz, Pz, Cz; 300–450 ms post feedback)	Larger P3 when selecting large magnitude than small magnitude option	–	–
Rigoni et al., 2010	*n* = 36 (12M), range 18–26 years, *SD* unavailable	Peak amplitude (CPz; 320–420 ms post feedback)	G > L; larger P3 for large gains and losses than small gains and losses	FRN peak amplitude (Fz; 260–360 ms post feedback)	G < L
Santesso et al., 2011	*n* = 30 (14M), *M* = 21.7 *SD* = 2.8, range 18–29 years	Peak amplitude (FCz, Cz, CPz; 300–500 ms post feedback)	larger P3 for large gains and losses than small gains and losses; no effect of valence was found	FRN peak amplitude (FCz, Cz, CPz; 200–400 ms post feedback)	G < L; larger amplitudes for small gains and losses than larger gains and losses; larger amplitudes for small gains compared to large gains, but no difference between large and small losses
Schuermann et al., 2012	*n* = 20 (5M), *M* = 29.5 *SD* = 8.9, range 21–52 years	Mean amplitude (CPz, Pz; 300–400 ms post feedback)	G < L; larger P3 for high-risk options than low-risk options	FRN peak amplitude (FCz, Fz; 200–400 ms post feedback)	G < L; larger for high-risk option between losses and gains, but this was not the case for low-risk option; amplitudes for gains reduced in the high-risk option compared to the low-risk option
Sun et al., 2015	*n* = 25 (0M), range 20–25 years, *M* and *SD* unavailable	Mean amplitude (Pz; 450–490 ms post feedback)	Detection of dishonest choice showed a larger P3 amplitude when participant believed they were playing against a computer as opposed to a human; no valence results reported	FRN mean amplitude (Fz; 250–280 ms post feedback)	G < L; smaller amplitudes for losses when participants believed the opponent was human compared to computer
Telpaz and Yechiam, 2014	*n* = 57 (30M), *M* = 23.4 *SD* = 2.3, range 19–27 years	Peak amplitude (Fz; 350–400 ms post feedback)	No significant associations	fERN peak amplitude (Fz; 250–300 ms post feedback)	No significant associations
Wang et al., 2015	*n* = 12 (7M), *M* = 22.6 *SD* = 1.6, range 20–25 years	Mean amplitude (FC3, FCz, FC4, C3, Cz, C4, CP3, CPz, CP4; 450–550 ms following risky stimulus)	Larger P3 amplitude when selecting the risky option compared to the ambiguous condition	–	–
Wang et al., 2017	*n* = 48 (24M), *M* = 20.2 *SD* = 1.6	Mean amplitude (Cz, CP1, CPz, CP2, Pz; 350–450 ms post feedback)	Larger P3 for large gains and losses than small gains and losses	FRN mean amplitude (Fz, FC1, FCz, FC2, Cz; 250–350 ms post feedback)	G < L; larger amplitude for large gains and losses than small gains and losses
Watts et al., 2017	*n* = 26 (14M), *M* = 19.4 *SD* = 1.9	Amplitude (mean or peak not specified; 9 electrodes around Cz; 250–500 ms post feedback)	Larger P3 for unexpected gains than expected gains; no valence results reported	FRN amplitude (mean or peak not specified; (9 electrodes around Cz; 203–352 ms post feedback)	G < L
Wu and Zhou, 2009	*n* = 19 (13M), range 22–26 years, *M* and *SD* unavailable	Peak amplitude (CP3, P3, CPz, Pz, CP4, P4; 250–600 ms post feedback)	G > L; larger P3 for large gains and losses than small gains and losses	FRN mean amplitude (F3, FC3, Fz, FCz, F4, FC4; 250–350 ms post feedback)	G < L; larger amplitude for small gains and losses than large gains and losses
Yang et al., 2013	*n* = 36 (12M), *M* = 22.0 *SD* = 2.2 years	Peak amplitude (CPz, Cz; 300–500 ms post feedback)	G > L; larger P3 for large gains and losses than small gains and losses	FRN peak amplitude (Fz, FCz, Cz, CPz, Pz; 200–400 ms post feedback)	No significant associations
Yang et al., 2015	*n* = 30 (16M), *M* = 22.1 *SD* = 2.3 years	Peak amplitude (CPz; 300–500 ms post feedback)	G > L; larger P3 for large gains and losses than small gains and losses	FRN peak amplitude (Fz, FCz, Cz; 200–400 ms post feedback)	No significant associations
Yu and Zhou, 2006	*n* = 20 (10M), *M* = 21.2 *SD* = 1.6 years	–	–	FRN mean amplitude and peak latency (Fz, FCz, Cz, CPz, Pz; 253–303 ms post feedback)	G < L
Yu and Zhou, 2009	*n* = 14 (7M), *M* = 21.4 *SD* = 1.5 years	–	–	FRN (200–300 ms post feedback) & ERN (300–500 ms post feedback); mean amplitudes (Fz, Pz, Cz)	FRN: G < L; larger amplitude for large gains and losses than small gains and losses ERN: Larger amplitudes for “to bet” than “not to bet” choice selection (at sites Fz & Cz); larger amplitudes for large gains and losses than small gains and losses (at sites Fz & Cz)
Zhang et al., 2013	*n* = 18 (9M), *M* = 21.6 *SD* = 2.5 years	Mean amplitude (FCz, Cz; 320–400 ms post feedback)	G > L; larger P3 following small gains and losses than large gains and losses	FRN mean amplitude (FCz, Cz; 240–300 ms post feedback)	G < L; larger amplitude following small gains and losses than large gains and losses
Zhang et al., 2014	*n* = 99 (81M), *M* = 20.5 *SD* = 2.3 years	Mean amplitude (FCz, FC1, FC2, Cz, C1, C2, CPz, CP1, CP2; 320–500 ms post feedback)	G > L, larger amplitudes for gain than neutral and ambiguous outcomes; larger P3 when switching from previous trial on the consecutive trial	FRN peak amplitude (FCz, FC1, FC2, Cz, C1, C2; 200–280 ms post feedback)	G < L, larger amplitudes for gain than neutral and ambiguous outcomes
Zhao et al., 2016	*n* = 25 (11M), *M* = 22.3, *SD* = 3 years	Mean amplitude (P3: Cz, CPz, Pz; time window unreported)	G > L; larger P3 following large gains and losses than small gains and losses	–	–
Zheng and Liu, 2015	*n* = 43 (21M), *M* and *SD* unavailable	Mean amplitude (CPz, Pz; 350–450 ms post feedback)	G > L; larger P3 following feedback from high-risk outcomes than low-risk outcomes	FRN mean amplitude (Fz, FCz; 250–350 ms post feedback)	Larger amplitude for high-risk selection compared to low-risk selection
Zheng et al., 2015	*n* = 16 (8M), *M* = 23, range 19–26 years, *SD* unavailable	Mean amplitude (CPz, Pz; 350–450 ms post feedback)	G > L; whether the outcome was a gain or loss was significant after high-risk options but not after low-risk options	FRN mean amplitude (Fz, FCz; 290–350 ms post feedback)	Larger following high-risk choices compared to low-risk choices in the gain context, while there was no difference of risk for the loss context
Zhu et al., 2014	*n* = 28 (16M), *M* and *SD* unavailable	–	–	FRN mean amplitude (Fz, FCz, Cz, CPz; 240–340 ms post feedback)	G < L at FCz; larger amplitudes in response to small losses than large losses
Zhu et al., 2015	*n* = 16 (9M), *M* = 22.6 *SD* = 0.8, range 22–24 years	P3 mean amplitude (CPz; 300–400 ms post feedback)	G > L	FRN peak amplitude (Fz, Cz; 220–320 ms post feedback)	No significant associations
Zhu et al., 2016a	*n* = 21 (11M), *M* = 21.4, *SD* = 0.8, range 20–14 years	–	–	FRN mean amplitude (Fz, Cz, FCz; 220–320 ms post feedback)	G < L
Zhu et al., 2016b	*n* = 21 (11M), *M* = 21.4, *SD* = 0.8, range 20–14 years	–	–	FRN mean amplitude (Fz, F1, F2, FC1, FC2, C1, C2, Cz; 220–320 ms post feedback)	G < L
Zhu et al., 2017	*n* = 27 (13M), *M* = 22.4, *SD* = 0.7, range 21–24 years	Mean amplitude (CPz, CP1, CP2; 320–420 ms post feedback)	G > L	FRN mean amplitude (Fz, FCz, Cz; 220–320 ms post feedback)	Valence results not reported
Zottoli and Grose-Fifer, 2012	*n* = 18 (18M), *M* = 24.1 *SD* = 1.2, range 22–26 years	–	–	FRN mean amplitude (F1, Fz, F2, FC1, FCz, FC2, C1, Cz, C2) and peak latency (Fz); 200–425 ms post feedback	G < L; significantly larger for small gains compared to large gains; latencies significantly longer for losses compared to gains; larger losses had longer latencies than small losses

*Major findings relate to associations between ERP componentry and risk (typically gain/loss) during gambling tasks. N, number; Mean (M) age and standard deviation (SD) of age is reported to one decimal place. Mean, standard deviation, and range of ages only presented if available. M, male; FRN, feedback related negativity; ERN, error-related negativity; MFN, medial frontal negativity; fERN, feedback error-related negativity. G > L, gain amplitudes are greater than loss amplitudes, G < L, gain amplitudes are smaller than loss amplitudes. Larger amplitude = more negative amplitude for the early error-detection component and more positive amplitude for the P3*.

* *Results are from the same study. Only experiment 1 self-execution trial results extracted for Ma et al. (2011)*.

+ *Risky stimulus is the presentation of stimuli with potential for loss outcomes or potential for loss of a large magnitude*.

∧ *Cue presentation refers to a cue stimulus indicating probability of gain/loss outcome*.

The following sections will review the results of the included studies, organized by task type and ERP component. The results from the early error detection components (i.e., FRN/ERN/MFN) are generally reported collectively when referring to amplitude and latency results in response to feedback. Given that the ERN can be separated into feedback ERN and response ERN, it is worth noting that all studies reporting the ERN report their major findings relative to feedback and not relative to response selection. Results for the ERN following response errors are reported separately in this review.

### Balloon analogous risk task (BART; 5 studies)

Three studies investigating the P3 in response to feedback from the BART found larger P3 components for loss compared to gain feedback (Euser et al., 2011; Kóbor et al., 2014; Ba et al., 2016). One study investigated effects of risk-taking and feedback, and described larger P3 amplitudes when selecting the high-risk compared to the low-risk option (Hassall et al., 2013). This study also reported larger P3 amplitudes for large gains and losses than small gains and losses. Three studies reported larger early error-detection components for loss than gain feedback (Euser et al., 2011; Mussel et al., 2015; Ba et al., 2016).

### Blackjack (3 studies)

Only one study reported larger P3 amplitudes in response to feedback signifying wins or losses of larger (compared to smaller) amounts of money (West et al., 2014a). This study also reported valence effects, such that P3 amplitudes were larger for busts (when a player's value was larger than 21), followed by losses (when the value was below 17), and wins (when the player's value was above 17 but also below 21). Another study showed the P3 amplitude to be larger for busts than for gains or losses, and also reported magnitude effects with larger P3 amplitudes following large gains/losses compared to small gains/losses (West et al., 2014b). This study also reported modulations of early error-detection component amplitudes, finding larger amplitudes for smaller gain/loss outcomes than larger outcomes (West et al., 2014b). Hewig et al. (2007) showed valence effects with larger early error-detection component amplitudes following loss compared to gain feedback. The study by Hewig et al. (2007) also reported that, the greater the probability of a gain outcome, the larger the amplitude following loss outcome.

### Cued learning gambling tasks (8 studies)

One of the six studies examining P3 amplitudes found larger amplitudes for monetary gain than for loss feedback (Deng et al., 2012). Two studies reported the opposite, with larger P3 amplitudes for loss than for gain feedback (San Martin et al., 2013, 2016), and two studies did not find differences between gain and loss feedback (Pfabigan et al., 2011a, 2012). One study examined gains and losses relative to whether the feedback was expected or unexpected, and whether the feedback was certain or uncertain (based on participants' estimated outcome probability), reporting larger P3 amplitudes for gains than losses in both the expected-uncertain and unexpected-certain feedback conditions (Kogler et al., 2017). Larger P3 amplitudes for loss than gain feedback was reported following expected-uncertain feedback in the same study. Two studies described larger P3 amplitudes elicited in response to both large gains and large losses compared to small gain/loss outcomes (San Martin et al., 2013, 2016). The two experiments by Pfabigan et al. (2011a) reported longer P3 latencies following loss feedback. Pfabigan et al. (2011b) provided gain/loss feedback using happy and sad faces, whereas Pfabigan et al. (2011a) provided feedback using words. One study examined the P3 relative to risk-taking, and found larger P3 amplitudes when selecting high-risk compared to low-risk options (He et al., 2013). All five studies examining early error-detection components reported larger amplitudes in response to loss feedback compared to gain feedback (Pfabigan et al., 2011a,b; San Martin et al., 2013, 2016; Kogler et al., 2017).

### Fruit gambling task (3 studies)

All three studies found larger P3 amplitudes in response to monetary gains as opposed to losses (Luo et al., 2011; Lole et al., 2013, 2015). Luo et al. (2011) differentiated between two types of loss feedback; near misses and full misses, reporting near misses to elicit larger P3 amplitudes than full misses. All three studies found a similar pattern for early error-detection component results, whereby loss feedback was associated with larger amplitudes than near losses (Luo et al., 2011), near gains (Lole et al., 2015), and gains (Lole et al., 2013). Lole et al. (2015) also investigated responses to feedback magnitude, reporting smaller early error-detection component amplitudes for large gains and losses compared to small gains and losses.

### Iowa gambling task (IGT; 11 studies)

Three of five studies reporting P3 valence effects identified larger P3 amplitudes for monetary gains compared to losses (Mapelli et al., 2014; Tamburin et al., 2014; Ba et al., 2016). One study presented neutral feedback (i.e., no gains or losses) in addition to gain and loss feedback, and reported larger P3 amplitudes for gains compared to neutral feedback (Azcárraga-Guirola et al., 2017). Another study categorized participants into two groups: favorable (participants who showed a pattern of advantageous deck selection) and undecided (participants who did not show a pattern of advantageous deck selection), and reported the favorable group to show smaller P3 amplitudes for gain outcomes, whilst P3 gain/loss differences were not identified in the undecided group (Giustiniani et al., 2015). Two studies found larger P3 amplitudes in response to feedback of larger magnitude gains and losses compared to smaller magnitude outcomes (Cui et al., 2013; Balconi et al., 2015). One study reported larger amplitudes for losses than gains when there was an 80% probability for a gain outcome and did not find any significant P3 amplitude differences for gains and losses with a 20% probability of a gain outcome (Fielding et al., 2017). Another study did not report any significant P3 associations (Schuermann et al., 2011). Relative to the early error-detection components, six out of the nine studies examining valence effects reported larger early error-detection component amplitudes for loss than gain feedback (Christie and Tata, 2009; Bianchin and Angrilli, 2011; Schuermann et al., 2011; Cui et al., 2013; Ba et al., 2016; Azcárraga-Guirola et al., 2017). Azcárraga-Guirola et al. (2017) also reported that neutral feedback produced larger early error-detection amplitudes than gain feedback. Two studies reported the opposite for the early error-detection components findings, showing larger amplitudes for gain as opposed to loss feedback (Mapelli et al., 2014; Tamburin et al., 2014). One study did not find any significant associations for the early error-detection components (Fielding et al., 2017). Two studies examined the P3 relative to risky decision-making: one study reported larger P3 amplitudes for risky compared to non-risky option selection (Christie and Tata, 2009), the other study found larger P3 amplitudes for high-risk bet selection over the right hemisphere and larger P3 for low-risk bet selection over the left hemisphere (Cui et al., 2013). Cui et al. (2013) also reported larger P3 amplitudes for large gains and losses, than small gains and losses.

### Single outcome gambling task (SOG; 4 studies)

Gain conditions, which were followed by a monetary gain, elicited larger P3 amplitudes and shorter P3 latencies, compared to loss conditions in the two studies examining P3 effects in the SOG (Kamarajan et al., 2009, 2010). Both studies also reported larger P3 amplitudes for gains of larger magnitudes than gains of smaller magnitudes. Kamarajan et al. (2010) also investigated latency differences, relative to the magnitude of the gain/loss feedback, reporting shorter latencies for small gain/loss outcomes than large gain/loss outcomes. Three studies examined early error-detection components following feedback (Masaki et al., 2006; Kamarajan et al., 2009; Onoda et al., 2010). Kamarajan et al. (2009) reported larger amplitudes for gain than loss feedback and shorter latencies for gain than loss feedback. In contrast, Onoda et al. (2010) found that amplitudes were larger following loss feedback compared to gain feedback, and larger amplitudes to larger magnitude gains and losses as compared to smaller gains and losses. Likewise, Masaki et al. (2006) reported larger amplitudes for loss feedback compared to gain feedback, however this only occurred for error outcomes (small monetary gain on gain outcomes and large monetary loss on loss outcomes were considered as error outcomes in this study).

### Two choice gambling tasks (46 studies)

#### P3 effects

Out of the 24 studies reporting P3 feedback effects for gains and losses, 19 studies reported larger P3 amplitudes for gains compared to losses (Wu and Zhou, 2009; Leng and Zhou, 2010, 2014; Polezzi et al., 2010; Rigoni et al., 2010; Ma et al., 2011; Nelson et al., 2011; Ibanez et al., 2012; Luo and Qu, 2013; Yang et al., 2013, 2015; Zhang et al., 2013, 2014; Mushtaq et al., 2015; Zheng and Liu, 2015; Zhu et al., 2015, 2017; Kardos et al., 2016; Zhao et al., 2016). Two studies did not find amplitude differences between gain and loss outcomes (Santesso et al., 2011; Telpaz and Yechiam, 2014), and three studies reported the opposite findings (Schuermann et al., 2012; Zheng et al., 2015; Endrass et al., 2016). Five studies did not report any valence effects (Oberg et al., 2011; Sun et al., 2015; Mushtaq et al., 2016; Wang et al., 2017; Watts et al., 2017). Nine out of the 11 studies reporting magnitude effects found consistent results with a larger P3 amplitude for larger magnitude outcomes than for smaller magnitude outcomes, irrespective of whether it was a gain or loss (Wu and Zhou, 2009; Polezzi et al., 2010; Rigoni et al., 2010; Santesso et al., 2011; Ibanez et al., 2012; Yang et al., 2013, 2015; Zhao et al., 2016; Wang et al., 2017), whilst the two other studies found the opposite effect, with larger P3 amplitude for smaller magnitude outcomes compared to larger magnitude outcomes (Zhang et al., 2013; Mushtaq et al., 2016). Watts et al. (2017) reported larger P3 amplitudes in response to unexpected gains compared to expected gains. Two studies explored P3 differences following feedback from high-risk (possible large magnitude gain/loss) and low-risk (possible small magnitude gain/loss) options (Zheng and Liu, 2015; Zheng et al., 2015). One study found the P3 amplitude to be larger after gains than losses following the high-risk but not following the low-risk option (Zheng et al., 2015), and the other study found a larger P3 for feedback from high-risk compared to low-risk outcomes (Zheng and Liu, 2015). Six studies assessed risky decision-making in relation to P3 amplitudes (Oberg et al., 2011; Schuermann et al., 2012; Rao et al., 2013; Wang et al., 2015; Zheng and Liu, 2015; Zheng et al., 2015). All the studies, except Zheng and Liu (2015), found larger P3 amplitudes when selecting the high-risk option compared to the low-risk option. In the study by Zheng and Liu (2015), the effect of loss and gain feedback was only significant following high-risk option selection.

#### Early error-detection component (FRN/ERN/MFN) effects

Out of the 43 studies measuring early error-detection components, 34 studies reported larger amplitudes for loss than for gain feedback (Gehring and Willoughby, 2002; Nieuwenhuis et al., 2004; Yu and Zhou, 2006, 2009; Goyer et al., 2008; Li et al., 2009; Wu and Zhou, 2009; Gu et al., 2010; Leng and Zhou, 2010, 2014; Marco-Pallares et al., 2010; Polezzi et al., 2010; Rigoni et al., 2010; Nelson et al., 2011; Santesso et al., 2011; Heitland et al., 2012; Ibanez et al., 2012; Schuermann et al., 2012; Zottoli and Grose-Fifer, 2012; Leicht et al., 2013; Luo and Qu, 2013; Zhang et al., 2013, 2014; Zhu et al., 2014, 2016a,b; Mushtaq et al., 2015, 2016; Sun et al., 2015; Endrass et al., 2016; Kardos et al., 2016; Kokmotou et al., 2017; Wang et al., 2017; Watts et al., 2017). Ma et al. (2011) reported the opposite effect, showing larger early error-detection component amplitudes in response to gain feedback compared to loss feedback. Marco-Pallares et al. (2010) showed a pronounced positivity in response to gains, whilst reporting a pronounced early error-detection component negativity for losses. The two experiments by Nieuwenhuis et al. (2004) reported different early error-detection component results, such that one reported larger early error-detection component amplitudes for loss than gain feedback, while the other study did not find an effect of outcome. The authors more specifically investigated the ERN, with one of their studies finding larger amplitude responses for only error trials compared to the correct trials. Four studies did not find significant amplitude differences between gain and loss feedback (Yang et al., 2013, 2015; Telpaz and Yechiam, 2014; Zhu et al., 2015), while four other studies did not report early error-detection component differences between gain and loss feedback (Oberg et al., 2011; Zheng and Liu, 2015; Zheng et al., 2015; Zhu et al., 2017). Relative to reward magnitude and feedback valence, larger amplitudes were observed in response to larger magnitude feedback than smaller magnitude feedback in two of 10 studies (Yu and Zhou, 2006; Wang et al., 2017). Five studies showed the opposite, with larger amplitudes in response to smaller magnitude feedback (Wu and Zhou, 2009; Santesso et al., 2011; Luo and Qu, 2013; Zhang et al., 2013; Zhu et al., 2014). Two studies also reported this pattern of results, however only for gain feedback (i.e., larger amplitudes in response to small than large gains; Li et al., 2009; Zottoli and Grose-Fifer, 2012). One study reported smaller amplitudes in response to large and small loss feedback compared to a large magnitude gain feedback (Ibanez et al., 2012). The study by Yu and Zhou (2009) investigated the feedback ERN following gamble/no gamble choice responses separately to the FRN following feedback in the same study, and reported larger ERN amplitudes following feedback when selecting to gamble than when selecting not to gamble. Three studies examined error-related negativity component amplitudes relative to risky decision-making (Oberg et al., 2011; Zheng and Liu, 2015; Zheng et al., 2015). Interestingly, Oberg et al. (2011) observed an early error-detection component following feedback for risky decision-making, but not following low-risk decision-making. Zheng et al. (2015) reported larger amplitudes for high-risk compared to low-risk option selection, whereas Zheng and Liu (2015) found larger amplitudes for high-risk compared to low-risk option selection for only gain outcomes (i.e., no difference in amplitude for high-risk and low-risk option selection for loss outcomes).

## Discussion

A total of 79 studies were identified that examined the P3 ERP component and early error-detection ERP components (here collectively referring to FRN, ERN, and MFN), elicited following risk-related decisions or task feedback. Results were largely consistent across studies. Larger P3 amplitudes followed feedback that indicated monetary gains compared to losses, and P3 amplitudes were larger for gains of a larger magnitude. Generally, larger (more negative) early error-detection components were found following loss feedback, as compared to gain feedback.

### The P3 component

P3 component amplitudes can be modulated by a range of factors: salience, task relevance, stimulus probability, surprise, novelty, and attention (Kok, 2001; Nieuwenhuis et al., 2005; Patel and Azzam, 2005). Recent findings suggest that P3 amplitude is linked to belief-updating: surprising information, which requires integration into current internal working models, tends to elicit larger amplitudes compared to non-surprising information (Mars et al., 2008; Bennett et al., 2015).

### The P3 component in relation to risky decision-making

Studies assessing P3 amplitudes in relation to decision-making consistently reported larger P3 amplitudes when participants selected the riskier option (e.g., Christie and Tata, 2009; Oberg et al., 2011; Schuermann et al., 2012; Cui et al., 2013; Hassall et al., 2013; He et al., 2013). These findings indicate that the variability in outcomes following a decision (i.e., the level of risk) can be evaluated rapidly following stimulus onset, which may influence P3 amplitudes through secondary mechanisms, as discussed below.

The P3 amplitude modulations can be explained by a greater emotional salience attribution to gaining rewards as opposed to losing, as the task goal is to gain as much reward as possible. By making the high-risk option more salient than the low-risk option, there is potential for a larger gain than in the low-risk option. However, choosing the high-risk option also entails losing a large amount, whereas selecting the low-risk option involves losing a small amount. Individuals tend to show greater sensitivity toward the possibility of losing compared to the possibility of gaining rewards (Tom et al., 2007). Selecting the high-risk option involves dealing with a greater level of threat, and the subjective influence of loss outcomes is believed to be twice that of gain outcomes (Tom et al., 2007). Therefore, the larger P3 may reflect the evaluation of the potential for a loss outcome.

Demonstrations of a larger P3 in response to the selection of the risky option can also be explained by Prospect Theory (Tversky and Kahneman, 1992). According to this theory, individuals maximize expected utility by choosing an option with the highest expected return. Decision-making requires the evaluation of the expected value, the value of an outcome and the probability of obtaining that outcome (Sambrook et al., 2012). The tendency to be loss aversive during risky decision-making can be attributed to the phenomenon that losses are perceived as more negative than the positive evaluation of equivalent gains. Hence, the possibility of loss from selecting a risky option could suggest increased deliberation and evaluation of the option (e.g., considering previous trial outcomes following the risky option), and this could be reflected by the larger P3 amplitudes for high-risk relative to low-risk option selection.

Another factor to consider when understanding these results is that, in the selected studies, selecting the high-risk option is likely to be more salient than in a real-world setting. This is because participants were aware that if they performed poorly, they nevertheless did not lose money. Participants might therefore have been more motivated to choose the risky option. This is in line with suggestions that the P3 amplitude is more affected by the overall task-relevance of the stimulus (i.e., maximize monetary net gain in this case) than by the valence of the stimulus (Nieuwenhuis, 2011). Taken together, these considerations suggest that selecting the high-risk option might have been of greater value to the decision-makers than the low-risk option in these specific decision-making contexts.

### The P3 in response to reward outcome feedback

The majority of reviewed studies found larger P3 amplitudes in response to positive feedback indicating a monetary gain. In many studies, participants were aware that their outcome could result in a monetary gain following the experiment based on their performance. Early work on the P3 has indicated high-value stimuli to produce larger amplitudes than low-value stimuli, with amplitudes being proportional to the amount of monetary reward associated with a stimulus outcome (Johnston et al., 1986).

Studies finding greater P3 amplitudes following positive feedback might be reflective of the affective (i.e., emotive) response when gaining positive rewards to be stronger than when obtaining a negative outcome (Luo and Qu, 2013; Leng and Zhou, 2014). These results suggest that such subjective affective evaluations take place from approximately 300 ms post feedback presentation. Furthermore, studies reporting larger P3 amplitudes for positive outcomes also found an effect of magnitude, such that larger gains elicited a larger P3 than smaller gains (Polezzi et al., 2010; Rigoni et al., 2010; Ibanez et al., 2012; Yang et al., 2015), supporting the idea that stimuli high in affect can elicit a large P3 amplitude. Individuals' tendency to have a positive bias could also be related to these findings, where individuals favor the integration of positive information as opposed to the integration of negative information during belief-updating (Chowdhury et al., 2014; Garrett and Sharot, 2014). Hence the larger P3 amplitude may reflect this integration of positive information into an individual's working memory.

Although it was more common to report larger P3 amplitudes for gain feedback than for loss feedback, inconsistencies across the studies may reflect differences in the paradigms employed. Larger P3 amplitudes in response to gain compared to loss feedback may be masked by the effect of numerical reward magnitude. For example, out of the 37 studies using the cued learning gambling task, fruit gambling task, Iowa gambling task, single outcome gambling task, or the two choice gambling task, and reporting P3 valence effects 29 studies reported larger amplitudes for gain feedback. In these studies, feedback outcome consisted of the presentation of valence and magnitude of outcome simultaneously, where the outcome was presented as a numerical value of the monetary amount won or lost on the trial. In the eight studies not showing this effect, there were differences in the paradigm, such that alternative feedback outcome was presented in addition to the actual feedback outcome following response selection (San Martin et al., 2013), the feedback was linked with participants' outcome expectation (Fielding et al., 2017; Kogler et al., 2017), or neutral feedback was presented as a potential feedback outcome in additon to gain and loss feedback (Azcárraga-Guirola et al., 2017). Two studies used faces as feedback stimuli along with the magnitude of the feedback outcome (Schuermann et al., 2012; Endrass et al., 2016), and another study presented the magnitude first, followed by the valence of feedback (Giustiniani et al., 2015). Interestingly, San Martin et al. (2016) provided feedback indicating the valence and magnitude of the outcome simultaneously; however, the outcome was presented as colored shapes, participants had to reason the valence and magnitude of the outcome based on the color of the shape and the number of these colored shapes, respectively. This paradigm is different to those showing the effect (i.e., larger P3 amplitudes for gain than loss feedback), as unlike the other studies, this study did not use a numerical value as feedback outcome, and the additional cognitive processes required to decode the feedback might have masked the P3 effects. The consistent finding in studies presenting numerical feedback regarding the outcome magnitude could be indicative of the P3 amplitude reflecting the processing of the value of numerical reward magnitudes.

Findings regarding feedback and the P3 differed for Blackjack and the BART. In Blackjack tasks, P3 amplitudes were larger following wins compared to losses, whereas the opposite pattern was found for the BART. The Blackjack gambling task is different to the other gambling paradigms as, for optimal performance, participants are constantly required to evaluate their current state in the game. This influences the decisions that the participant makes, that is, whether to stay with the current set of cards or select another card (West et al., 2014a). In this task, losses might lead to larger belief-updates (Endrass et al., 2016; Kardos et al., 2016), and therefore to larger P3 amplitudes following losses. Similarly, the BART also taps into constant updating of working memory during the task, as participants are able to infer that, as the task continues, the chance of the balloon bursting increases (Kóbor et al., 2014). The findings from the study by Ba et al. (2016) support this idea as this study employed both the IGT and the BART and reported opposite P3 feedback effects from the same participant sample.

Greater salience for positive feedback compared to negative feedback may also be induced by the experimental manipulation in the tasks. Negative feedback did not result in a negative outcome for participants as none of the studies required reimbursement from participants for the amount of money they had lost, if the total gambling outcome resulted in a loss of money. This is different to positive feedback in studies that paid participants their winnings, where participants received a monetary reward proportionate to their performance on the task. Hence, in these studies the reward feedback is more salient compared to studies where participants did not receive their winnings. However, the fact that the results were largely comparable between studies in which a monetary bonus was paid out based on performance and those studies in which no such bonus was paid suggests that another factor could account for the findings. One such factor could be the task goal: participants were instructed to gain as many rewards as possible during the task; hence, they might have been likely to attribute salience to positive feedback as it is task-relevant.

Larger P3 amplitudes were also found for unexpected feedback compared to expected feedback (e.g., Pfabigan et al., 2011a). This fits with the hypothesis that the P3 indexes surprise (Mars et al., 2008; Endrass et al., 2016), and is in accordance with oddball paradigms where larger P3 amplitudes are found in response to unexpected and less frequent stimuli (Donchin, 1981; Polich, 2007; Yuan et al., 2007). The unexpected feedback may reflect a revision of the mental representation, a belief-update, of the specific decision that leads to the feedback on the gambling trials (Bennett et al., 2015; Endrass et al., 2016; Kardos et al., 2016).

Multiple brain regions have been associated with the P3 (i.e., P3b): the inferior parietal lobe in the posterior parietal cortex and the temporoparietal junction (TPJ) are commonly identified as being implicated in its generation (Linden, 2005). The posterior parietal cortex and TPJ activation are linked with goal-directed attention and stimulus processing respectively, suggesting these areas are connected with evaluation of risky stimuli and feedback from risky decision-making. Additionally, the anterior cingulate cortex (ACC) is activated during the integration of information to internal working models to predict future events (O'Reilly et al., 2013), supporting studies reporting a link between ACC activation and the P3 (Smith et al., 1990; Wang et al., 2005).

It is apparent that the P3 provides an important index for risky decision-making and evaluation of feedback from decision-making. Synthesis of the findings from the studies examining the P3 in response to risky decision-making has highlighted the influence of numerical magnitudes in modulating the P3 amplitude when processing feedback, and has demonstrated the P3 to be implicated in the evaluation of risky response selection. Additionally, P3 amplitudes following feedback may reflect belief-updating, where the integration of information into one's working memory is required to shape decision-making under risk. Feedback-evoked P3 amplitudes may serve as an important indicator of learning in risk-related tasks with uncertain outcomes (e.g., Bennett et al., 2015).

### Early error-detection components

The early error-detection components are influenced by feedback valence and the magnitude of outcomes (i.e., FRN, MFN, feedback ERN), as well as by the detection of unexpected outcomes and errors (i.e., response ERN). Larger (more negative) amplitudes are generally elicited in response to negative feedback, negative feedback of a larger magnitude, and in response to the detection of endogenous response errors (Holroyd et al., 2003; Ullsperger et al., 2014).

The majority of reviewed studies reported more negative early error-detection components following loss as opposed to gain feedback (i.e., only four out of the 59 studies reporting valences differences, found the opposite effect). This suggests that the evaluation of feedback outcome occurs within 250 ms and implies these early error-detection components to be more susceptible to negative feedback and aversive outcomes (Oliveira et al., 2007). Early error-detection components may reflect evaluation of events as being relatively favorable or unfavorable (Holroyd et al., 2004; Hajcak et al., 2006). For example, under conditions where only neutral and positive feedback are presented, the neutral feedback is considered unfavorable when the task goal is to maximize rewards. Sensitivity to negative feedback can help guide future behavior through learning which actions are likely to produce negative feedback, allowing these actions to be avoided in the future. For example, a study by Cohen and Ranganath (2007) demonstrated that the amplitude of the FRN could predict behavior on subsequent trials. The authors found larger amplitudes to loss feedback when the subject chose an alternate response on the subsequent trial, compared to when the same response was selected. This suggests that participants changed their behavior based on prior performance and experienced conflict following unfavorable feedback.

The early error-detection components are also sensitive to expectancy, such that, when engaging in gambling tasks, individuals tend to expect positive feedback, regardless of the probability of obtaining a positive outcome (Oliveira et al., 2007). This discrepancy between expectation and outcome is believed to result in larger early error-detection components with these components indexing the violation of expectations instead of merely evaluating feedback valence (Mapelli et al., 2014). Traditionally, only the ERN was considered to result from reward prediction errors, however studies have reported findings consistent with the FRN component also being evoked in response to surprising events (Oliveira et al., 2007; Chase et al., 2011; Talmi et al., 2013). Hence, it is apparent that early error-detection components reflect surprise or expectation violations instead of feedback valence alone (Hauser et al., 2014). These considerations support the early Conflict-Monitoring theory, which postulates that the FRN is responsible for the detection of errors. However, there are models suggesting that the ERN might be sensitive not only to error detection *per se* (Falkenstein et al., 1990; Gehring et al., 1993), but more broadly reflect the degree of experienced conflict (e.g., Botvinick et al., 2001) and action monitoring (Vidal et al., 2000). Hence, coinciding instead with Reinforcement Learning theory, which suggests differential dopaminergic activity relative to expected (decrease in dopamine activity) and unexpected (increase in dopamine activity) outcomes, can guide future decision-making (Holroyd and Coles, 2002).

Dopamine signals can influence the initiation and regulation of behavior through communication with the ACC, leading to the integration of reward related information to improve task performance (Walsh and Anderson, 2012), through learning stimulus-outcome contingencies. These findings suggest evaluation (i.e., good vs. bad) and feedback confirming or disconfirming predictions of potential feedback outcomes are linked with the ACC. Neuroimaging studies have demonstrated increased activation in the ACC following monetary loss and reduced reward feedback (O'Doherty et al., 2001; Bush et al., 2002), suggesting reward processing to be associated with this area and supporting the idea that early error-detection components originate from the ACC (Holroyd and Coles, 2002; Nieuwenhuis et al., 2004).

Expectation is more likely to play a role in tasks that involve learning reward probabilities. For example, in the Iowa Gambling Task, participants implicitly learn which of the four decks are advantageous and which decks are disadvantageous as the task progresses. Likewise, in cued learning gambling tasks, participants are able to predict outcomes based on cue associations with outcome probability. In these tasks, the amplitudes of the early error-detection component could have been influenced more so by expectation than by reward valence. Hewig et al. (2007) examined expectation effects and found the probability of a gain outcome to be predictive of the size of the ERN amplitude in Blackjack following feedback. Hence, the type of task can influence the magnitude of the evoked early error-detection component.

Based on the findings from the early error-detection components, it is apparent that the valence of the feedback (i.e., positive or negative) and the subjective probability of the feedback outcome plays a role in the elicited amplitude, suggesting that these components are capable of indexing risk-related decision-making relative to decision outcomes.

Overall, the findings from both the P3 and the early error-detection components have demonstrated that risky decision-making in the economic context elicit consistent ERP responses. While most of these tasks are designed to operationalize economic risk-taking as it might occur in the real world, it remains unclear how exactly risk-taking in a laboratory setting translates to risk-taking in real-world contexts.

### Limitations and future directions

The findings of this review should be interpreted with the following limitations. All the reviewed studies employed a gambling paradigm, which is specific to the field of economic risk-taking (Blais and Weber, 2006). Significantly, employing a gambling task was not an inclusion criteria, rather, we could only identify gambling tasks using our search. Results from these designs may not generalize to all forms of risk-taking. Future studies could investigate the psychophysiology of risk-taking in other fields, such as in recreational and social scenarios. This would help identify how the P3 and the early error-detection components are related to risky decision-making in other domains of risk; in particular whether a larger P3 is always elicited in response to risk-taking, or whether a larger negativity for the early error-detection component is always observed in response to negative feedback. For feedback processing following risky decisions, results might potentially differ depending on the domain of risk. One of the selected studies integrated a social aspect to gambling, where individuals competed against a friend or stranger, and reported P3 and early-error detection component responses similar to their own when observing a friend gambling (Ma et al., 2011). However, when the individuals engaged in the gambling task (i.e., competed with others), results suggested a less pronounced amplitude difference between gain and loss outcomes for observation of the friend's performance compared to when only observing the game between the friend and stranger (Ma et al., 2011). This highlights that the inclusion of social risk factors might indeed change the pattern of results and could potentially shed light on the cognitive mechanisms driving the observed amplitude differences.

Only the commonly reported ERP components, in this case the P3 and FRN/ERN/MFN, were selected to be reviewed. Other ERP components were also mentioned in the selected studies, such as reward positivity, however these components were not explored here given the small number of studies reporting effects for these components relative risky decision-making and feedback.

Inconsistent results for the P3 component were obtained from gambling tasks that involve planning during risk-taking (e.g., in Blackjack) and tasks that have a different feedback format (e.g., using face stimuli, or presenting numerical outcome and feedback valence separately), suggesting that the P3 amplitude may primarily reflect belief-updating and numerical processing of risk, respectively. Hence, future studies could systematically examine the ERP responses to these cognitive processes in relation to risk-taking. Quantitative modeling of single trial ERP component amplitudes (e.g., Bennett et al., 2015) across a range of risk, expectation, and reward outcome conditions could help dissociate the contribution of each factor to P3 amplitudes. In contrast to the P3, early error-detection ERP responses following feedback appeared to not vary markedly across tasks. Given that no one ideal paradigm exists to measure risk, it might be worthwhile to incorporate different aspects of risk (e.g., risky response selection, feedback valence, expectation, magnitude of monetary outcomes), as identified in this review, in one paradigm and systematically study the effects of manipulating those aspects on the ERP components in the same participants. The seven different categories of gambling tasks employed by the reviewed studies (with additional variances to risky stimulus presentation and feedback presentation), give rise to ambiguity in determining the specific manipulations that lead to the magnitude of the evoked ERP components.

The tasks employed by the reviewed studies varied regarding the probability of a given outcome. For example, in the BART, participants are required to pump the balloon for as long as possible before the balloon bursts as this increases the number of points one can gain during the task. However, the risk of the balloon bursting increases incrementally with each balloon pump, giving rise to balloon pumps later on in the trial acquiring greater risk potential compared to balloon pumps early on in the trial. This task feature is similar to Blackjack. On a task such as the two-choice gambling task, risk-taking is involved when one selects to gamble the high monetary value as opposed to the low monetary value on each trial. The level of risk-taking in this case does not increase with each subsequent trial (i.e., selecting the high-risk gamble on subsequent trials is no more risky than the previous trial). Hence, the differences in stimulus frequencies may influence the level of risk-taking a participant engages in, consequently resulting in differences in their ERP responses. In the case of the BART, risk-taking is increased with a subsequent balloon pump, whereas in a two-choice gambling task, the level of risk-taking remains consistent as the trials progress.

This review has established that several ERP component modulations are replicable across a range of gambling tasks and experimental designs. Investigating individual differences in these component modulations may be able to determine whether propensity for risk-taking can be predicted by the magnitude of these ERP effects. Studies using functional magnetic resonance imaging (fMRI) have already demonstrated individual differences in neural activity related to risk processing. One study, investigating the decision-making phase in a two-choice gambling task, reported that individuals with a greater tendency to be loss aversive showed larger blood-oxygen-level dependent (BOLD) signals in the ventral striatum and prefrontal cortex for both loss and gain outcomes (Tom et al., 2007). This suggests that risk-seeking and risk-avoidant individuals might differ in their sensitivity to negative and positive feedback. Another study, also using fMRI, examined the sensitivity to risk anticipation in individuals categorized as having high and low risk preferences (i.e., risk-seekers and risk avoiders respectively) in a binary gambling task, and reported that risk-seekers showed reduced activation in the ventral striatum and anterior insula when anticipating a risk outcome compared to risk-avoiders (Rudorf et al., 2012).

The early error-detection components, which have not been differentiated here (reflecting how they are commonly conflated in the risk literature) are believed to index at least two distinct cognitive processes: one is the immediate response to error commission, which occurs around 100 ms following an erroneous action, and the other process is triggered by feedback for an action, which occurs approximately 250 ms following feedback (Holroyd et al., 2003). However, in many of the selected studies, the time windows and terminology were used interchangeably, which prevented clear dissociations between components in this review. We suggest that future studies employ clearer operationalized definitions of these components, allowing for a more refined picture of the FRN, ERN, and MFN in relation to risk-taking to emerge. Additionally, a meta-analysis of the selected studies was not conducted as many studies did not report effect sizes for ERP component amplitude differences. Future studies should report effect sizes and measures of uncertainty such as confidence intervals, which are often much more informative than *p*-values.

## Conclusions

There are consistent effects across studies linking larger P3 amplitudes to increased risk-taking and positive feedback to risky bets, and linking larger early error-detection component amplitudes to negative feedback following risky decision-making. Both the P3 and early error-detection components show consistent associations with risk-taking and feedback. These associations appear to be reliable effects found in the context of the majority of risk-related decision-making tasks. We hope that these findings inform future research in the risk field, especially in terms of moving beyond gambling tasks into other risk-related domains, and employing identified experimental manipulation effects to further untangle risk-related neural mechanisms. Review findings also have utility in other cognitive domains outside of risk-related decisions and feedback, as these ERP components are not specific to risk.

## Author contributions

All persons who meet authorship criteria are listed as authors, and all authors certify that they have participated sufficiently in the work to take public responsibility for the content, including participation in the concept, design, analysis, writing, or revision of the manuscript. DC and HK were involved in the conception and design of study. Data acquisition was through DC and MG. DC, DF, SB, and HK were involved in analysis and interpretation of data. The manuscript was written by DC with initial review by HK. Further critical revision and final approval of the manuscript was by HK, DF, SB, and MG.

### Conflict of interest statement

The authors declare that the research was conducted in the absence of any commercial or financial relationships that could be construed as a potential conflict of interest.
